# Validation of Identified Susceptible Gene Variants for New-Onset Diabetes in Renal Transplant Recipients

**DOI:** 10.3390/jcm8101696

**Published:** 2019-10-16

**Authors:** Hyeon Seok Hwang, Kyung-Won Hong, Jin Sug Kim, Yang Gyun Kim, Ju Young Moon, Kyung Hwan Jeong, Sang Ho Lee, The Korean Organ Transplantation Registry Study Group

**Affiliations:** 1 Division of Nephrology, Department of Internal Medicine, College of Medicine, Kyung Hee University, Seoul 130-702, Korea; hwanghsne@gmail.com (H.S.H.); jinsuk0902@hanmail.net (J.S.K.); apple8840@hanmail.net (Y.G.K.); kidmjy@hanmail.net (J.Y.M.); aprilhwan@naver.com (K.H.J.); 2 Division of Healthcare Innovation, TheragenEtex Bio Institute Co., Ltd., Suwon 443-721, Korea; kyungwon.hong@therabio.kr

**Keywords:** new onset diabetes after renal transplantation, single nucleotide polymorphisms, renal transplantation

## Abstract

Genome-wide association studies (GWAS) and candidate gene approaches have identified single nucleotide polymorphisms (SNPs) associated with new-onset diabetes after renal transplantation (NODAT). We evaluated associations between NODAT and SNPs identified in previous studies. We genotyped 1102 renal transplant recipients from the Korean Organ Transplantation Registry (KOTRY) database; 13 SNPs were assessed for associations with NODAT (occurring in 254 patients; 23.0%), within one year after transplantation. The frequency of the T allele at *KCNQ1* rs2237892 was significantly lower in patients with NODAT compared to control patients (0.30 vs. 0.39; *p* = 8.5 × 10^−5^). The T allele at rs2237892 was significantly associated with decreased risk of NODAT after adjusting for multiple variables, compared to the C allele (OR 0.63, 95% CI 0.51–0.79; *p* = 5.5 × 10^−5^). Dominant inheritance modeling showed that CT/TT genotypes were associated with a lower risk for development of NODAT (OR 0.56, 95% CI 0.42–0.76; *p* = 2.0 × 10^−4^) compared to the CC genotype. No other SNPs were associated with NODAT. Our study validated the protective effect of T allele at *KCNQ1* rs2237892 on the development of NODAT in a large cohort of renal transplant recipients. Our findings on susceptibility variants might be a useful tool to predict NODAT development after renal transplantation.

## 1. Introduction

Development of new-onset diabetes after renal transplantation (NODAT) is a common complication in patients that have undergone transplantation. The cumulative incidence of NODAT is approximately 15%–30% at 1-year post-transplantation, and the annual incidence of NODAT is approximately 4%–6% [1,2,3]. This metabolic disorder induces a worse cardiovascular risk profile and results in a three-fold risk of cardiovascular morbidity [4,5]. In addition, NODAT is associated with a 1.5- to 3-fold risk of allograft loss and results in a 10%–20% reduction in long-term patient survival [1,6,7]. The accumulated health-care cost is also considerable, with an estimated cost of US $21,500 per new patient with diabetes in the second year after transplantation [8]. Therefore, NODAT is a critical burden of recipient care and a major clinical challenge for the longevity and survival of renal allograft patients.

The risk of developing NODAT is associated with several clinical factors, including the recipient age, BMI, use of tacrolimus and corticosteroid, acute rejection, hepatitis C virus, cytomegalovirus infection, autosomal dominant polycystic kidney disease, and hypomagnesemia [1,9,10,11,12,13,14,15]. However, evidence suggests an increased incidence of NODAT despite the identification of clinical risk factors and the effort to mitigate the risk [16]. As current strategies have limited effectiveness in preventing NODAT, genetic risk stratification emerges as a key approach to address this problem.

Several studies have shown genetic predisposition as a risk factor for the development of NODAT. Genetic polymorphism studies on NODAT led to the identification of several candidate genes, derived from genome-wide association studies (GWAS) for type 2 diabetes [17,18]. Commonly evaluated genetic determinants included genes involving carbohydrate metabolism, insulin secretion, and insulin resistance [19]. In addition, genes that encode inflammatory cytokines correlated with type 2 diabetes and were also associated significantly with NODAT [20]. More recently, GWAS showed that genes involved in *β*-cell apoptosis are associated with the development of NODAT [21,22]. However, candidate gene approaches included only a few individuals with NODAT, leading to inconsistent results, and the significant genes identified in GWAS are not replicated in independent cohorts. Therefore, these limitations severely interrupt the development of prevention strategies against NODAT.

This study aimed to verify the association of previously identified genetic polymorphisms with NODAT in a large nationwide prospective cohort. We selected 17 single nucleotide polymorphisms (SNPs) on susceptibility loci and evaluated the effects of these independent SNPs on the risk of developing NODAT.

## 2. Materials and Methods

### 2.1. Study Population

The study population was selected from the Korean Organ Transplantation Registry (KOTRY), which is a prospective, multicenter, nationwide cohort study that includes transplantation information in Korea. Thirty-two representative national hospitals and transplantation centers participated in KOTRY. Recipients were enrolled consecutively upon undergoing a transplantation procedure and followed up accordingly from July 2014 to December 2018. The registry accumulated data on individual patients including demographics, comorbidities, laboratory data, induction and maintenance of the immunosuppressive regimen, and several other types of events. Our study was reviewed and approved by the Institutional Review Board of each transplantation center. All patients provided written informed consent before enrollment in the study.

Blood samples from 1826 patients were stored for genotyping and screened using the KOTRY database. The following patients were excluded: Renal transplant recipients with established diabetes (*n* = 503), patients followed up for less than one year (*n* = 107), non-functioning graft at one-year follow-up (*n* = 32), incomplete record of medical or laboratory findings (*n* = 65), missing information on human leukocyte antigen (HLA) typing (*n* = 2), and others (*n* = 15). In total, finally, 1102 patients were enrolled for this study.

### 2.2. Selection of SNPs and Genotyping

We conducted an extensive literature review for published variants that were significantly associated with NODAT in renal transplant recipients. We evaluated SNPs, which showed top-ranked associations with NODAT in individual studies. We selected seventeen SNPs that were significantly associated with NODAT from GWAS or well-established association studies of NODAT [18,20,21,22].

Blood samples (3 mL each) were collected in tubes containing RBC lysis solution. The blood sample from each study participant was centrifuged to obtain white blood cells. Genomic DNA was extracted from white blood cells using a DEX^TM^ II genomic DNA extraction kit (Intron, Sungnam, Korea). DNA samples were stored at −80 °C before analysis. Quality of stored DNA samples was evaluated using agarose gel electrophoresis to confirm sample integrity. SNPs were genotyped from these DNA samples using TaqMan-based QuantStudio OpenArray^®^ (Life Technologies, Carlsbad, CA, USA). DNA from patients and controls was randomly transferred into 96-well plates and genotyped using a blinded method. The call rates for genotyping of the SNPs were >98%.

### 2.3. Data Collection and Definition

We collected the following baseline patient characteristics at the time of transplantation: Age, gender, body mass index (BMI), relevant comorbid conditions, information on human leukocyte antigen (HLA), blood typing, desensitization, and induction and maintenance of the immunosuppressive regimen. Laboratory data were collected at baseline and regularly followed up. Clinical events were identified, including diabetes, the occurrence of biopsy-proven acute rejection, all-cause graft loss, and patient death or follow-up loss.

The primary outcome was the evaluation of SNP impact on the risk of developing NODAT within the first year after transplantation. Based on the definition of the American Diabetes Association, NODAT was diagnosed when fasting blood sugar was higher than 126 mg/dL six months after transplantation, or when insulin or oral hypoglycemic agents were required for treatment [23]. The control group consisted of renal transplant recipients who did not meet NODAT criteria during the follow-up period.

### 2.4. Statistical Analysis

Continuous variables were presented as the mean ± standard deviation. Allelic frequencies were analyzed using a chi-squared test between the two groups. Student’s t-tests and chi-squared tests were used to evaluate between-group differences for continuous and categorical variables, respectively. For all SNPs, minor allele frequency (MAF), compliance with Hardy-Weinberg equilibrium (HWE), linkage disequilibrium analysis, and the association between rs2237892 and NODAT in different genetic models were assessed using SNPstats software (https://www.snpstats.net/start.htm). A multivariate logistic regression model was used to investigate the confounding effects of clinical variables significantly associated with NODAT and SNP associations. We included clinical covariates according to their weights in univariate testing, and we included clinically fundamental parameters. The confounders used in this analysis were recipient age, recipient sex, BMI, HLA mismatch number, desensitization in HLA incompatibility, ABO incompatibility, use of tacrolimus, use of steroids, biopsy-proven acute rejection, donor age, and deceased donor. Bonferroni correction was used in the association analysis when multiple comparisons were performed. We used multiple inheritance models, including codominant (major allele homozygotes vs. heterozygotes vs. minor allele homozygotes), dominant (major allele homozygotes vs. minor allele homozygotes plus heterozygotes), recessive (major allele homozygotes plus heterozygotes vs. minor allele homozygotes), and log-additive (major allele homozygotes vs. heterozygotes vs. minor allele homozygotes) models. Statistical analyses were performed using SPSS for Windows software (version 20.0; SPSS, Chicago, IL, USA). The significance level was set at *p* < 0.05.

## 3. Results

### 3.1. Baseline Clinical Characteristics and SNP Information

The incidence of NODAT in this study population was 23.0% (254/1102 patients). Baseline characteristics of recipients are summarized in Table 1. Transplant recipients who developed NODAT were significantly older, tended to be male, and had higher BMI scores than those who did not develop NODAT. Donor age in the NODAT group was significantly higher than in the control group. Desensitization treatment for HLA incompatibility was used more frequently in the control group. There was no difference between the two groups in the incidence of biopsy-proven acute rejection, or the use of tacrolimus or steroids as maintenance immunosuppressant treatments.

We excluded *AGMAT* rs11580170 from further analysis because it was in strong linkage disequilibrium with *DNAJC16* rs7533125 (*r*^2^ = 0.99). Of rs1494558 and rs2172749 in *IL7R*, only rs2172749 was analyzed, because these SNPs were also in linkage disequilibrium (*r*^2^ = 0.98). Of the 15 SNPs tested, 14 were consistent with HWE (*p* > 0.05). While *DNAJC16* rs7533125 violated HWE in the control group (*p* = 0.037), minor allele frequency (MAF) did not deviate from that of the East Asian population [24]. Therefore, we included *DNAJC16* rs7533125 in the genetic association test. We additionally excluded *TCF7L2* rs7903146 and *NPPA* rs198372 in the association test, because MAF was less than 0.05 (frequency of T allele at *TCF7L2* rs7903146, 0.02; and frequency of A allele at *NPPA* rs198372, 0.01).

### 3.2. Allelic Frequency and Association between SNPs and NODAT

The allele frequencies of the genetic polymorphisms in the NODAT and control groups are summarized in Table 2. The allelic frequency of the T allele at *KCNQ1* rs2237892 was significantly lower in patients with NODAT compared to that in the control group (0.30 vs. 0.39; *p* = 8.5 × 10^−5^). The C allele at *CDKAL1* rs10946398 had a higher frequency in the NODAT group, with marginal statistical significance (0.52 vs. 0.47; *p* = 0.080).

We examined the genetic association between SNPs and NODAT in an allele-specific pattern (Table 3). Univariate analyses showed that the T allele at *KCNQ1* rs2237892 was significantly associated with decreased risk of NODAT (odds ratio (OR) 0.66, 95% confidence interval (CI) 0.53–0.82; *p* = 1.3 × 10^−4^). The C allele at *CDKAL1* rs10946398 was associated with a 1.2-fold higher risk for development of NODAT (95% CI 0.98–1.46; *p* = 0.078). However, none of the other SNPs evaluated in this study (*ATP5F1P6* rs10484821, *DNAJC16* rs7533125, *CELA2B* rs2861484, *CASP9* rs2020902, *NOX4* rs1836882, *INPP5A* rs4394754, *IL7R* rs2172749, *IL17R* rs4819554, *IL17RB* rs1025689, *IL17RB* rs1043261, and *PLXDC1* rs72823322) were significantly associated with NODAT. The association between *KCNQ1* rs2237892 and NODAT was enhanced when evaluated using multivariate logistic regression analysis (OR 0.63, 95% CI 0.51–0.79; *p* = 5.5 × 10^−5^). However, no other SNPs were significantly associated with NODAT in the multivariate logistic regression analysis.

### 3.3. Genotype Distribution and Association between KCNQ1 rs2237892 and NODAT

We tested the effect of *KCNQ1* rs2237892 genotype on NODAT using a multiple inheritance model as shown in Table 4). In the codominant model, the TT genotype at rs2237892 was associated with the lowest risk for development of NODAT, compared to the CC genotype (OR 0.41, 95% CI 0.25–0.67; *p* = 4.7 × 10^−4^). In the dominant model, the CT/TT genotype was also associated with a reduced risk for development of NODAT (OR 0.56, 95% CI 0.42–0.76; *p* = 2.0 × 10^−4^). The T allele significantly reduced the risk of NODAT compared to the CC genotype in the log-additive model. However, no significant differences were observed in the recessive model with Bonferroni correction.

## 4. Discussion

In the present study, using samples from a large cohort of renal transplant recipients, we examined the association of 13 SNP pairs and candidate genes for risk of NODAT development. Of the studied variants, there was a significant difference in the frequency of the T allele at *KCNQ1* rs2237892 between the NODAT and control groups, and this allele showed an independent association with NODAT. The TT and CT genotypes of *KCNQ1* rs2237892 were associated with a significantly reduced risk for development of NODAT in codominant, dominant, and log-additive models. These findings suggested that the genetic variant of *KCNQ1* is a significant contributor to the development of NODAT in renal transplant recipients.

Although NODAT results from the combined effect of insulin resistance and *β*-cell dysfunction, several recent studies have shown that *β*-cell dysfunction is the main contributing factor for the development of NODAT [3,25,26]. *KCNQ1* rs2237892 and *CDKAL1* rs10946398 were identified as a susceptibility gene for type 2 diabetes in GWAS, and each of these genes is associated with *β*-cell dysfunction [27,28,29,30,31]. Previous studies with type 2 diabetes risk genes suggested an association between *KCNQ1* rs2237892 and NODAT [19]. Our study also validated that variant rs2237892 of the T allele was associated with decreased risk for development of NODAT compared to the C allele. Similarly, *CDKAL1* rs10946398 was also associated with NODAT, as reported in a study that used a candidate gene approach in patients who underwent transplantation [18,19]. However, our data did not confirm this association. These findings suggested that *KCNQ1* is a more robust and influential indicator of *β*-cell dysfunction in renal transplant recipients.

*KCNQ1* encodes a subunit of the voltage-gated K^+^ channel, which is expressed in pancreatic islets [32]. In the *KCNQ1*-overexpressing pancreatic *β*-cell line, the density of the K+ current increased significantly and affected the pancreatic cell membrane action potential [33]. Therefore, *KCNQ1* overexpression contributes to impairment of glucose-stimulated insulin secretion, and a specific *KCNQ1* blocker also stimulates insulin secretion [34]. In addition, allelic mutation of *KCNQ1* results in up-regulation of the neighboring gene, cyclin-dependent kinase inhibitor 1C, which encodes a cell cycle inhibitor and leads to reduction in pancreatic *β*-cell mass [35]. Therefore, we suggest that variant *KCNQ1* induces impaired *β*-cell function and reduced *β*-cell mass, and this biological function could be a potential underlying mechanism for the association between *KCNQ1* variants and increased risk for NODAT development.

Three types of *KCNQ1* SNPs were evaluated as potential risk factors for the development of NODAT in Spanish patients who received kidney transplants from deceased donors [17]. *KCNQ1* rs2237895, rs2237892, and rs8234 were genotyped, and SNP rs2237895, but not rs2237892, was found to be associated with an increased risk for development of NODAT in the first year after transplantation. This apparent discrepancy could be due to the allele frequencies of these SNPs. The T allele frequency at rs2237892 was reported to be 0.34–0.36 in the East Asian population, but only 0.04–0.08 in the European population [36]. Consequently, lower MAF at rs2237892 was not significantly associated with NODAT in Spanish transplant recipients. Therefore, we suggest that different genetic backgrounds should be considered when attempting to determine the risk of development of NODAT using *KCNQ1* genetic variants as indicators.

In a recent GWAS, numerous variants were found to be associated with risk for the development of NODAT [21]. *ATP5F1P6*, *CELA2B*, *CASP9*, *NOX4*, and *INPP5A* were identified as risk genes in Caucasian renal transplant recipients. These genetic variants were implicated in *β*-cell apoptotic pathways, but not insulin resistance, suggesting that *β*-cell apoptosis was a critical component of NODAT pathogenesis. However, our study did not find a significant association between NODAT and any SNPs from this GWAS. Three possible factors might explain this inconsistency: First, the *β*-cell apoptotic pathways could be a weak contributor to the development of NODAT in Asian compared to Caucasian recipients of a renal transplant. Second, a different definition of NODAT phenotype might have resulted in dissimilar findings in the two studies. Third, the limited sample size in the GWAS may have less power to detect significant associations [37].

Inflammatory cytokines are involved in insulin action and insulin secretion. An SNP within the gene encoding the IL-7R chain was found to be associated with type 1 diabetes mellitus [38,39]. Moreover, our previous study showed that genetic variants of *IL-7R*, *IL-17R*, and *IL-17RB* were associated significantly with NODAT [14]. However, the relevant SNPs of these interleukin genes were not associated with NODAT in the exploratory GWAS analysis or the secondary verification analysis [17]. Furthermore, our validation study also showed no meaningful differences in allele frequencies. These findings suggested that the effects of interleukin gene polymorphisms on the risk for development of NODAT were inconclusive, and further studies are necessary to obtain precise results.

The present study had a few limitations. As data regarding family history of type 2 diabetes were not available, the association between family history and the development of NODAT could not be evaluated. In addition, the effect of BMI and weight gain after transplantation was not included in our analysis. Finally, we did not perform an oral glucose tolerance test or HbA1c estimation before kidney transplantation. Therefore, patients with prediabetes might have been included in our study.

In conclusion, our validation study showed a significant association between *KCNQ1* rs2237892 and development of NODAT in a large cohort. Our results suggest that *KCNQ1* might play a crucial role in the pathogenesis of NODAT following renal transplantation. *KCNQ1* variants might be a useful tool to predict NODAT development in renal transplant recipients, and help screen for patients at a higher risk for NODAT.

## Figures and Tables

**Table 1:** Baseline demographics and characteristics of the study population.

	NODAT (*n* = 254)	Controls (*n* = 848)	*p*
Recipient			
Age (years)	52.2 ± 10.4	45.1 ± 12.0	<0.001
Male (%)	152 (59.8)	445 (52.5)	0.039
Dialysis duration (months)	63.7 ± 72.0	59.1 ± 67.0	0.384
BMI	23.2 ± 3.3	22.3 ± 3.2	<0.001
Donor			
Age (years)	48.6 ± 12.5	45.7 ± 12.9	0.001
Male (%)	136 (53.5)	467 (55.1)	0.668
Deceased (%)	108 (42.5)	310 (36.6)	0.086
HLA mismatch number	3.4 ± 1.7	3.2 ± 1.7	0.083
Desensitization for HLA incompatibility (%)	42 (16.5)	189 (22.3)	0.048
ABO incompatibility (%)	18 (7.1)	75 (8.8)	0.377
Anti-thymocyte globulin induction (%)	64 (25.2)	196 (23.1)	0.493
Tacrolimus (%)	252 (99.2)	831 (98.0)	0.191
Steroid (%)	252 (99.2)	845 (99.6)	0.367
Biopsy-proven acute rejection (%)	35 (13.8)	96 (11.3)	0.288

BMI = body mass index; and NODAT = new-onset diabetes after renal transplantation.

**Table 2:** Allele frequencies of polymorphisms previously associated with NODAT.

Gene	SNP	Chr: Position	Minor Allele	MAF			
				All	NODAT	Control	*p*
*CDKAL1*	rs10946398	6:20660803	C	0.48	0.52	0.47	0.080
*KCNQ1*	rs2237892	11:2818521	T	0.37	0.30	0.39	8.5 × 10^−5^
*ATP5F1P6*	rs10484821	6:139547773	C	0.33	0.32	0.33	0.583
*DNAJC16*	rs7533125	1:15557249	C	0.07	0.07	0.07	0.747
*CELA2B*	rs2861484	1:15486170	T	0.07	0.07	0.06	0.599
*CASP9*	rs2020902	1:15507865	G	0.04	0.04	0.04	0.930
*NOX4*	rs1836882	11:89498993	C	0.27	0.28	0.27	0.587
*INPP5A*	rs4394754	10:132529558	T	0.09	0.10	0.09	0.632
*IL7R*	rs2172749	5:3585516	C	0.40	0.40	0.40	0.976
*IL17R*	rs4819554	22:17084145	G	0.43	0.45	0.42	0.256
*IL17RB*	rs1025689	3:53849695	C	0.45	0.47	0.44	0.226
*IL17RB*	rs1043261	3:53865249	T	0.10	0.11	0.10	0.256
*PLXDC1*	rs72823322	17:39130161	G	0.21	0.22	0.21	0.185

NODAT = new onset diabetes after renal transplantation; Chr = chromosome; MAF = minor allele frequency; and SNP = single nucleotide polymorphism.

**Table 3:** Allele-based incidence and risk of NODAT.

Gene	SNP	Allele	Crude		Adjusted *	
			OR (95% CI)	*p*	OR (95% CI)	*p*
*CDKAL1*	rs10946398	C (vs. A)	1.20 (0.98, 1.46)	0.078	1.22 (0.98, 1.50)	0.070
*KCNQ1*	rs2237892	T (vs. C)	0.66 (0.53, 0.82)	1.3 × 10^−4^	0.63 (0.51, 0.79)	5.5 × 10^−5^
*ATP5F1P6*	rs10484821	C (vs. T)	0.94 (0.76, 1.17)	0.583	0.96 (0.77, 1.20)	0.726
*DNAJC16*	rs7533125	C (vs. T)	0.94 (0.65, 1.37)	0.756	0.96 (0.65, 1.43)	0.855
*CELA2B*	rs2861484	T (vs. G)	1.11 (0.76, 1.62)	0.607	1.07 (0.71, 1.60)	0.751
*CASP9*	rs2020902	G (vs. A)	0.98 (0.61, 1.56)	0.933	0.92 (0.56, 1.50)	0.733
*NOX4*	rs1836882	C (vs. T)	1.06 (0.85, 1.33)	0.588	1.02 (0.80, 1.29)	0.904
*INPP5A*	rs4394754	T (vs. C)	1.08 (0.78, 1.51)	0.635	1.08 (0.76, 1.54)	0.657
*IL7R*	rs2172749	C (vs. G)	1.00 (0.81, 1.22)	0.976	1.07 (0.86, 1.33)	0.535
*IL17R*	rs4819554	G (vs. A)	1.12 (0.92, 1.37)	0.255	1.09 (0.88, 1.35)	0.415
*IL17RB*	rs1025689	C (vs. G)	1.13 (0.93, 1.38)	0.226	1.15 (0.93, 1.41)	0.204
*IL17RB*	rs1043261	T (vs. C)	1.21 (0.87, 1.67)	0.252	1.21 (0.86, 1.71)	0.265
*PLXDC1*	rs72823322	G (vs. A)	0.85 (0.66, 1.09)	0.190	0.84 (0.65, 1.10)	0.199

NODAT = new onset diabetes after renal transplantation; CI = 95% confidence interval; OR = odds ratio; and SNP = single nucleotide polymorphism. *Adjusted for recipient age, recipient sex, BMI, HLA mismatch number, desensitization in HLA incompatibility, ABO incompatibility, use of tacrolimus, use of steroids, biopsy-proven acute rejection, donor age, and deceased donor.

**Table 4:** NODAT incidence and risk of *KCNQ1* rs2237892 in multiple inheritance models.

Model	Type	N (%)		OR (95% CI) *	*p*
		NODAT	Control		
Codominant	CC	128 (50.4)	317 (37.4)	Reference	
	CT	101 (39.8)	395 (46.6)	0.62 (0.45, 0.85)	2.8 × 10^−3^
	TT	25 (9.8)	136 (16.0)	0.41 (0.25, 0.67)	4.7 × 10^−4^
Dominant	CC	128 (50.4)	317 (37.4)	Reference	
	CT/TT	126 (49.6)	531 (62.6)	0.56 (0.42, 0.76)	2.0 × 10^−4^
Recessive	CC/CT	229 (90.2)	712 (84.0)	Reference	
	TT	25 (9.8)	136 (16.0)	0.53 (0.33, 0.84)	0.0051
Log-additive	-			0.63 (0.51, 0.79)	<1.0 × 10^−4^

NODAT = new onset diabetes after renal transplantation; CI = 95% confidence interval; and OR = odds ratio. * Adjusted for recipient age, recipient sex, BMI, HLA mismatch number, desensitization in HLA incompatibility, ABO incompatibility, use of tacrolimus, use of steroids, biopsy-proven acute rejection, donor age, and deceased donor.
